# Causal effect of psychiatric disorders on epilepsy: A two‐sample Mendelian randomization study

**DOI:** 10.1002/brb3.2939

**Published:** 2023-03-01

**Authors:** Gongfei Li, Minghui Wang, Meiqi Zheng, Xiao Liu, Tingting Yu, Jiechuan Ren, Qun Wang

**Affiliations:** ^1^ Department of Neurology Beijing Tiantan Hospital Capital Medical University Beijing China; ^2^ China National Clinical Research Center for Neurological Diseases, Beijing Tiantan Hospital, Capital Medical University Beijing China; ^3^ Beijing Tongren Eye Center Beijing Tongren Hospital Capital Medical University Beijing China; ^4^ Department of Anesthesiology Peking Union Medical College Hospital Chinese Academy of Medical Sciences and Peking Union Medical College Beijing China; ^5^ Beijing Institute for Brain Disorders, Collaborative Innovation Center for Brain Disorders Capital Medical University Beijing China

**Keywords:** epilepsy, Mendelian randomization, psychiatric disorder, risk factors

## Abstract

**Background:**

This study aims to explore the relationship between psychiatric disorders and the risk of epilepsy using Mendelian randomization (MR) analysis.

**Methods:**

We collected summary statistics of seven psychiatric traits from recent largest genome‐wide association study (GWAS), including major depressive disorder (MDD), anxiety disorder, autism spectrum disorder (ASD), bipolar disorder (BIP), attention deficit hyperactivity disorder (ADHD), schizophrenia (SCZ), and insomnia. Then, MR analysis estimates were performed based on International League Against Epilepsy (ILAE) consortium data (*n*_case_ = 15,212 and *n*_control_ = 29,677), the results of which were subsequently validated in FinnGen consortium (*n*_case_ = 6260 and *n*_control_ = 176,107). Finally, a meta‐analysis was conducted based on the ILAE and FinnGen data.

**Results:**

We found significant causal effects of MDD and ADHD on epilepsy in the meta‐analysis of the ILAE and FinnGen, with corresponding odds ratios (OR) of 1.20 (95% CI 1.08–1.34, *p* = .001) and 1.08 (95% CI 1.01–1.16, *p* = .020) by the inverse‐variance weighted (IVW) method respectively. MDD increases the risk of focal epilepsy while ADHD has a risk effect on generalized epilepsy. No reliable evidence regarding causal effects of other psychiatric traits on epilepsy was identified.

**Conclusions:**

This study suggests that major depressive disorder and attention deficit hyperactivity disorder may causally increase the risk of epilepsy.

## Graphical abstract

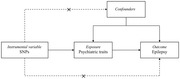

## 1 INTRODUCTION

Epilepsy is one of the most common serious brain conditions and is defined as recurrent unprovoked seizures. This condition affects over 70 million people worldwide (Singh & Trevick, 2016; Thurman et al., 2018). A sizeable body of evidence indicates that cerebral infection, brain tumors, stroke, traumatic brain injury, and autoimmune disorders are risk factors for epilepsy, but the etiology remains unclear in approximately 50% of new‐onset epilepsy cases (Neligan et al., 2012).

It is reported that approximately 50% of adults with active epilepsy have at least one comorbid disorder, which are present in about one fourth of patients with newly diagnosed epilepsy (Giussani et al., 2021; Keezer et al., 2016). One in three patients with epilepsy may experience a psychiatric disorder in the course of their life, mainly including mood and anxiety disorders, attention deficit hyperactivity disorder (ADHD) and psychosis (Kanner, 2016). The prevalence of psychiatric disorders is higher in patients with epilepsy both before and after the diagnosis of epilepsy (Berg et al., 2017; Dagar & Falcone, 2020). However, it remains challenging to measure the causal relationship between psychiatric disorders and epilepsy independent of possible confounding factors. No randomized controlled trial or large prospective study has elucidated this potential causal effect. If the risk effect of psychiatric disorders on the development of epilepsy is identified, more comprehensive and powerful management may be properly conducted and more mechanisms behind them may be discovered in terms of this spectrum of comorbidities and epilepsy.

Mendelian randomization (MR) is an epidemiological approach that uses genetic variation as a natural experiment to investigate the causal associations between potential risk factors and outcomes in observational data (Emdin et al., 2017). MR, simulating randomized controlled trials, is less likely to be affected by confounding and reverse causality biases than observational studies. Considering the power of the causation evidence, MR sits at the interface of randomized controlled trial and observational studies (Davies & Holmes, 2018). Many studies have been increasingly conducted using this useful method in other fields, while few analyses regarding epilepsy have been reported (Allman et al., 2018).

In this study, we performed a two‐sample MR study to evaluate the causal relationship between psychiatric disorders and epilepsy for the first time. Seven psychiatric traits were enrolled from the recent largest genome‐wide association study (GWAS), including major depressive disorder (MDD), anxiety disorder, autism spectrum disorder (ASD), bipolar disorder (BIP), ADHD, schizophrenia (SCZ), and insomnia.

## 2 MATERIALS AND METHODS

### 2.1 Study design

**FIGURE 1:**
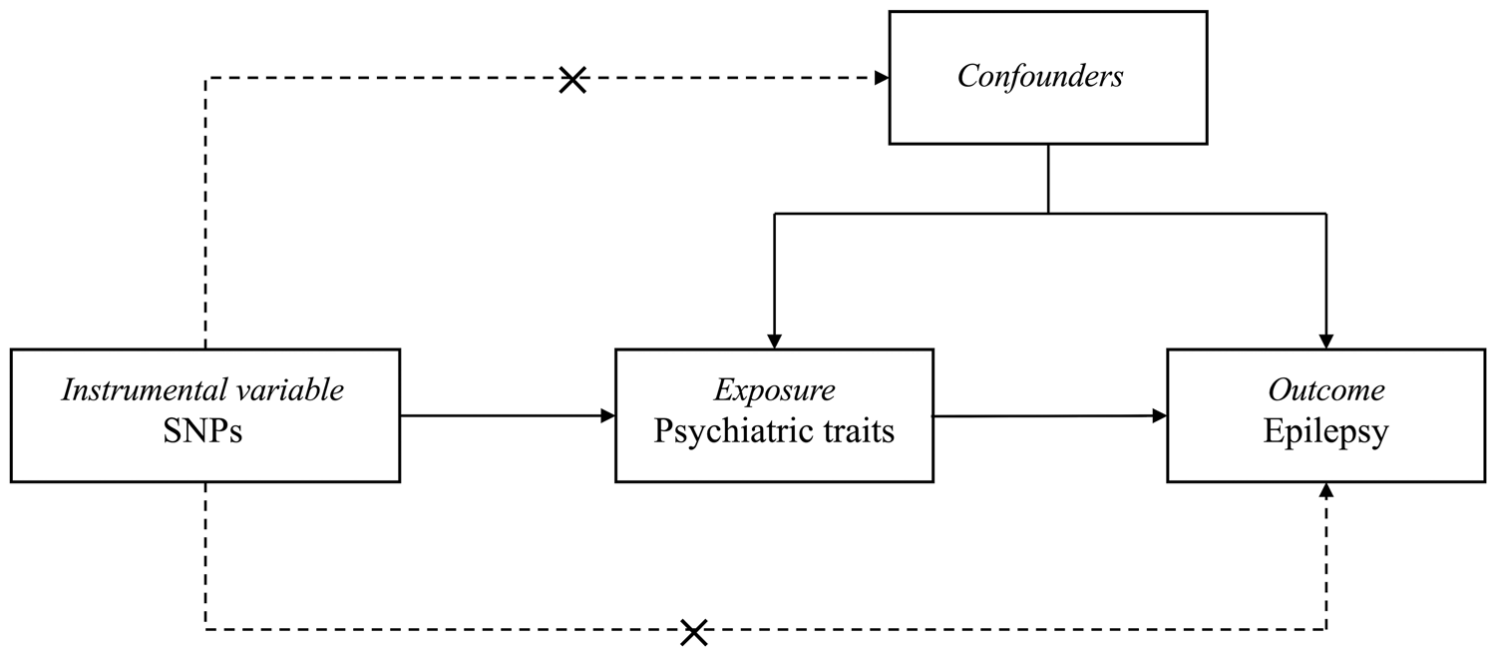
Conceptual framework for the Mendelian randomization analysis of the causal effect of psychiatric traits on epilepsy. The design is based on the assumption that the genetic variants are associated with psychiatric traits, but not with confounders, and affect epilepsy only through psychiatric traits. SNPs, single‐nucleotide polymorphisms.

We conducted a two‐sample MR analysis to investigate the causal effect of seven psychiatric traits on the risk of epilepsy, following the recommendations of Strengthening the Reporting of Observational Studies in Epidemiology Using Mendelian Randomization (STROBE‐MR) (Skrivankova et al., 2021). This MR study relies on three assumptions: (1) the instrumental variable (IV) is associated with the exposure (the relevance assumption); (2) the instrument variable shares no common cause with the outcome (the independence assumption); and (3) the instrument variable only affects the outcome through the exposure (the exclusion restriction assumption), which are presented in Figure 1 (Davies & Holmes, 2018). All data in this study were published by multiple GWASs; ethics approval and patient consent can be found in the original studies.

### 2.2 Data sources and genetic instruments

GWAS summary statistics of seven psychiatric traits were derived from published studies with large sample sizes of European ancestry. The definitions of the seven psychiatric traits are listed in Table S1. GWASs of ADHD, ASD, MDD, BIP, and SCZ were based on data from the Psychiatric Genomics Consortium (PGC). PGC is the largest international consortium of scientists dedicated to conducting meta‐ and mega‐analyses of genomic‐wide genetic data, with a focus on psychiatric disorders (Sullivan et al., 2018). For insomnia and anxiety disorders, we obtained the genetic associations from GWAS based on the UK Biobank data (Table 1) (Rusk, 2018).

**TABLE 1:** Data sources of the instrumental SNPs

Psychiatric traits	Population	Sample size (cases/controls)	Data source	Number of significant associated SNPs
ADHD	Europeans	20,183/35,191	PGC	7
ASD	Europeans	18,382/27,969	PGC	22^a^
MDD	Europeans	135,458/344,901	PGC	23
BIP	Europeans	20,352/31,358	PGC	11
SCZ	Europeans	33,640/43,456	PGC	55
Insomnia	Europeans	462,341 in all^b^	UKB	29
Anxiety disorder	Europeans	6410/456,523	UKB	4^a^

^a^The significance threshold of these two psychiatric traits was *p* < 1 × 10^−5^.

^b^The trait, insomnia, was categorical ordered that answered the question “Do you have trouble falling asleep at night or do you wake up in the middle of the night?” designed in the questionnaire on sleep. The categorical variable was coded as never/rarely, sometimes, usually, and we recorded the number of all samples.

Abbreviations: ADHD, attention deficit/hyperactivity disorder; ASD, autism spectrum disorder; MDD, major depressive disorder; BIP, bipolar disorder; SCZ, Schizophrenia; UKB, the UK Biobank; PGC, the Psychiatric Genomics Consortium.

We obtained GWAS summary statistics for all kinds of epilepsy (*n*_case_ = 15,212 and *n*_control_ = 29,677), generalized epilepsy (*n*_case_ = 3769 and n_control_ = 29,677), and focal epilepsy (*n*_case_ = 9671 and *n*_control_ = 29,677) from the International League Against Epilepsy (ILAE) Consortium (The International League Against Epilepsy Consortium on Complex Epilepsies, 2019). Seizure phenotype and epilepsy syndrome were classified according to the classification and terminology outlined by the ILAE. The ILAE consortium is a multi‐ancestry genome‐wide association meta‐analysis of epilepsy and seven different epilepsy subtypes testing single‐nucleotide polymorphisms (SNPs) for association with epilepsy. More details were published in the original study, including information about case enrolment, demographic characteristics, quality control, and study power.

To validate our analysis, summary statistics of the epilepsy data set from FinnGen consortium (*n*_case_ = 6260 and *n*_control_ = 176,107) were collected. The diagnosis of epilepsy in FinnGen was defined by G40 in the International Classification of Disease (ICD), 10th version. The genotype data were obtained from Finnish biobanks, and digital health record data were obtained from Finnish health registries. Further details of all these consortiums can be found at ^https://gwas.mrcieu.ac.uk.16^

**FIGURE 2:**
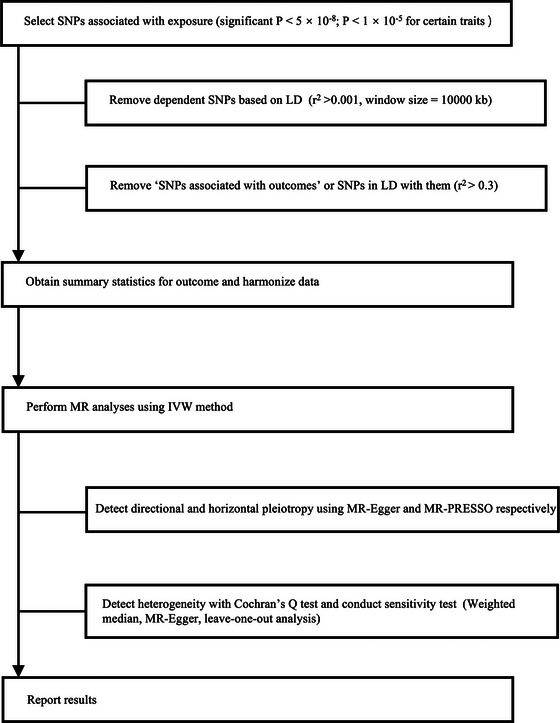
The analysis flow chart of this study. SNPs, single‐nucleotide polymorphisms; LD, linkage disequilibrium; IVW, inverse‐variance weighted; MR‐PRESSO, Mendelian Randomization Pleiotropy Residual Sum and Outlier.

As shown in Figure 2, SNPs strongly associated with exposure were extracted as candidate IVs at the genome‐wide significance threshold (*p* < 5 × 10^−8^) for each psychiatric trait, except for anxiety disorder and ASD, the SNPs of which were selected with the threshold of 1 × 10^−5^. Then, dependent SNPs with high linkage disequilibrium (LD) were removed from the candidate IV set based on the following parameter (*r*2 > 0.001, window size = 10,000 kb). Subsequently, among the candidate SNPs, outcome‐related SNPs (SNPs associated with epilepsy) were also removed according to the basic study assumption. Finally, we excluded certain candidate SNPs that had high LD (*r*2 > 0.3) with outcome‐related SNPs. For all exposures, we filtered the instruments for *F* statistics > 10 to mitigate potential effects of weak instrument bias. The remaining SNPs were used as valid IVs to perform MR analysis. The number of valid IVs for all exposure‐outcome pairs is listed in Table 1.

### 2.3 Statistical analysis

In this study, the inverse‐variance weighted (IVW) method was used to calculate estimates of associations between psychiatric traits and epilepsy. Briefly, the IVW method was performed assuming all SNPs were valid IVs with balanced pleiotropy. To determine whether unbalanced pleiotropy causing bias exists, the intercept from the MR–Egger regression was calculated to test directional pleiotropy (*p* < .05 infers that SNPs influence the outcome through different biological pathways other than exposure).

To further control for horizontal pleiotropy, the MR pleiotropy residual sum and outlier (MR‐PRESSO) method was applied. MR‐PRESSO was based on the IVW regression framework and detected IVs of horizontal pleiotropy as outliers in the regression. In particular, MR‐PRESSO implements a global test based on the leave‐one‐out approach and an outlier test to detect specific SNPs with horizontal pleiotropy. In addition, multiplicative random effects IVW was performed if we found potential heterogeneity across individual SNPs, which was estimated by Cochran's *Q* statistic (*p* < .05 was considered statistically significant).

Several sensitivity analyses were performed to validate the robustness of the IVW method, which was robust to pleiotropy. The first method was weighted median regression, which required that at least 50% of the weight for the MR analysis comes from valid instruments. The second method was MR‐Egger regression, which can help detect and adjust directional pleiotropy. Moreover, weighted mode and simple mode were also used as supplementary sensitivity analyses.

Finally, we performed a meta‐analysis of ILAE and FinnGen data to strengthen the power of the MR analysis. All estimates were reported with *p* values, and odds ratios with 95% confidence intervals for epilepsy risk were scaled to one SD increase in genetically associated psychiatric traits. All analyses were conducted with R 4.1.3, TwoSampleMR, and MR‐PRESSO packages.

## 3 RESULTS

### 3.1 Causal association of psychiatric traits with epilepsy in ILAE

The IVW MR analysis showed correlations between certain psychiatric traits and epilepsy based on the ILAE data. Estimates of the causal effects of seven psychiatric traits on epilepsy are presented in Table 2. MDD and ADHD were found to have suggestive risk effects on epilepsy, while BIP showed a protective effect on epilepsy. The corresponding effect sizes from the IVW method were OR = 1.17 (95% CI 1.03−1.33, *p* = .018), 1.09 (1.01−1.17, *p* = .018), and 0.93 (0.88−0.98, *p* = .009) for MDD, ADHD and BIP, respectively. Although some other estimates between these three traits and epilepsy did not reach statistical significance, the trends were in the same direction. The associations between each SNP and individual psychiatric traits and the risk of epilepsy are shown in the supplementary material (Tables S2‐S8).

**TABLE 2:** Associations of 7 psychiatric traits with epilepsy in MR analyses (based on ILAE data)

Exposure	IVW method			Weighted median method			MR‐Egger regression		
	OR	95% CI	*p*	OR	95% CI	*p*	OR	95% CI	*p*
ADHD^*^	1.09	1.01–1.17	.018	1.06	0.97–1.17	.180	1.21	0.90–1.63	.265
ASD	0.99	0.92–1.06	.691	0.99	0.92–1.07	.803	0.89	0.62–1.26	.514
MDD^*^	1.17	1.03–1.33	.018	1.22	1.05–1.42	.011	2.16	0.99–4.70	.066
BIP^*^	0.93	0.88–0.98	.009	0.93	0.87–1.01	.074	0.95	0.67–1.33	.760
SCZ	1.00	0.96–1.04	.903	1.00	0.96–1.04	.906	0.96	0.76–1.21	.721
Insomnia	0.74	0.54–1.01	.056	0.69	0.45–1.05	.085	1.13	0.18–6.99	.896
Anxiety disorder	8.34× 10^−4^	4.61×10^−7^ −1.51	.064	1.35× 10^−2^	3.41×10^−6^ −53.42	.308	2.66× 10	1.24×10^−5^ −5.67×10^−5^	.411

*Psychiatric traits that has significant *p* value (*p* < .05) in IVW method.

Abbreviations: CI: confidence interval; IVW: inverse‐variance weighted; OR: odds ratio.

In sensitivity analyses, Cochran's *Q*‐derived *p* was calculated from MR‐Egger regression (*p* = .055, .627, .808, respectively) and showed no evidence of heterogeneity for the instrumental variables of these three exposures. Additionally, no horizontal pleiotropy was found, with an insignificant intercept from the MR‐Egger test (*p* = .132, .507, .910, respectively) and no outliers identified from the MR‐PRESSO test. The results of leave‐one‐out sensitivity analyses suggested that the causal associations between psychiatric traits and epilepsy were not affected by any individual SNP (Figures S1, S3, and S5). More details of the sensitivity test are listed in the Table S12.

### 3.2 Causal association of psychiatric traits with epilepsy in FinnGen and meta‐analysis

Further validation was conducted based on data from the FinnGen consortium, and meta‐analysis of the ILAE and FinnGen was subsequently performed. Similarly, the IVW method showed a significant causal effect of MDD on epilepsy, with an OR of 1.31 (95% CI 1.05–1.63, *p* = .017). However, no causal or protective association was found between ADHD, BIP and epilepsy in the independent FinnGen sample. For the MR‐analysis of MDD and epilepsy, MR‐Egger regression and MR‐PRESSO suggested no heterogeneity (*p* = .667) or pleiotropy (*p* = .148 for MR‐Egger intercept and *p* = .603 from MR‐PRESSO). The leave‐one‐out sensitivity analyses suggested that the causal associations between psychiatric traits and epilepsy in FinnGen were not affected by any individual SNP (Figures S2, S4, and S6). The associations between each SNP and individual psychiatric traits and the risk of epilepsy are shown in the supplementary material (Tables S9‐S11).

**FIGURE 3:**
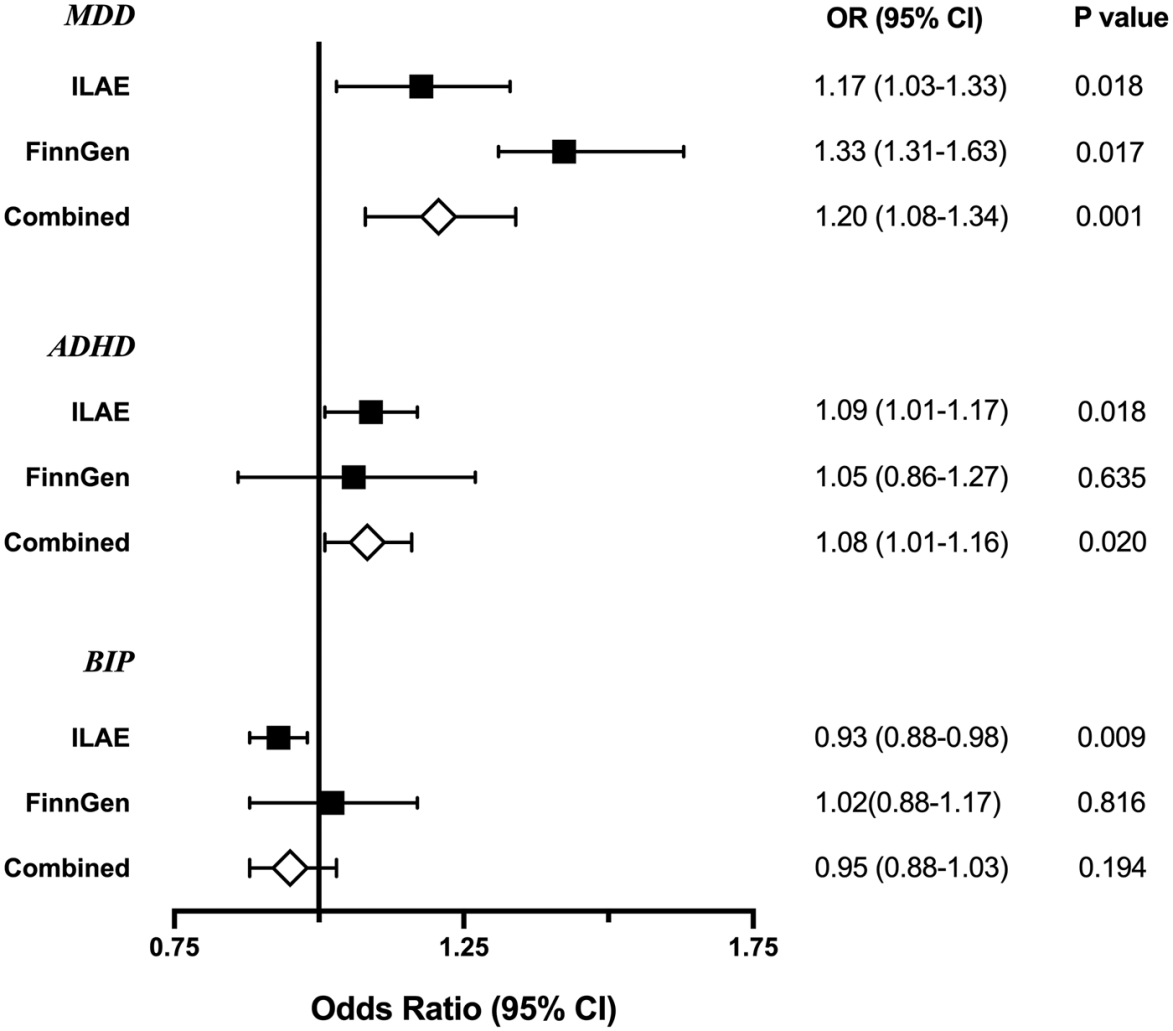
Associations of three psychiatric traits (MDD, ADHD, and BIP) with epilepsy based on the IVW method in International League Against Epilepsy (ILAE), in FinnGen, and a meta‐analysis of both data sets.

The meta‐analysis of the ILAE and FinnGen showed that genetically associated MDD (OR = 1.20, 95% CI 1.08−1.34, *p* = .001) and ADHD (OR = 1.08, 95% CI 1.01−1.16, *p* = .020) had a suggestive causal effect on epilepsy and increased the risk of epilepsy as shown in Figure 3. There was no heterogeneity between the MR analysis enrolled in our meta‐analysis (*p* = .384, .725 for MDD and ADHD, respectively).

### 3.3 Causal association of MDD and ADHD with focal epilepsy and generalized epilepsy

**FIGURE 4:**
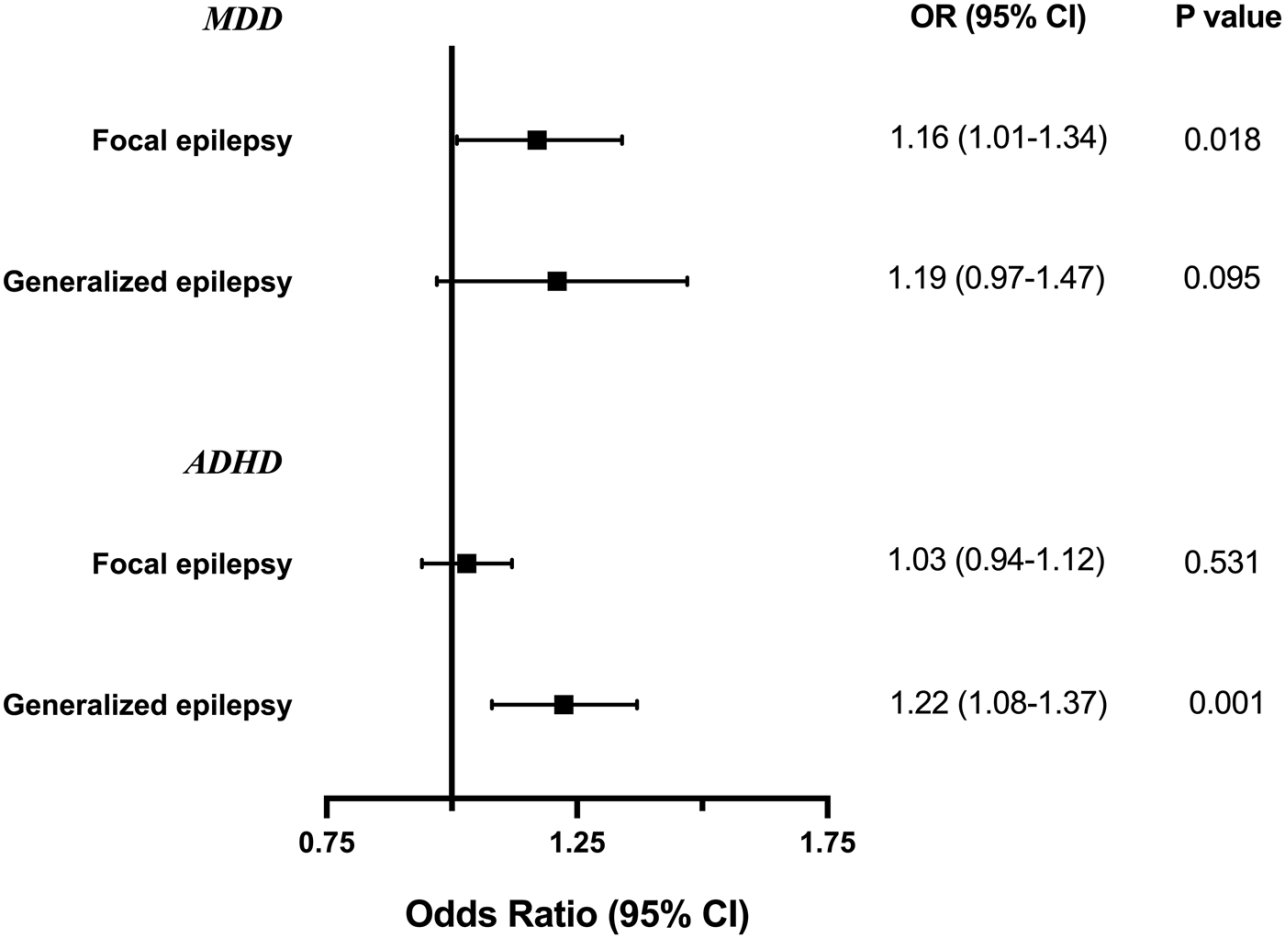
Associations of major depressive disorder and attention deficit hyperactivity disorder with focal and generalized epilepsy based on the IVW method in International League Against Epilepsy (ILAE).

The results showed that MDD had a causal association with focal epilepsy (OR = 1.16, 95% CI 1.01−1.34, *p* = .039) but no risk effect on generalized epilepsy (OR = 1.19, 95% CI 0.97−1.47, *p* = .095). Conversely, a causal effect was found between ADHD with only generalized epilepsy (OR = 1.22, 95% CI 1.08−1.37, *p* = .001) instead of focal epilepsy (OR = 1.03, 95% CI 0.94−1.12, *p* = .531). These results were shown in Figure 4. No heterogeneity or pleiotropy was identified in the sensitivity analyses.

## 4 DISCUSSION

In this study, we investigated the causal effect of seven psychiatric traits on epilepsy using MR analysis for the first time. A risk effect was found in MDD and ADHD. Specifically, MDD increased the risk of focal epilepsy, while ADHD provoked the development of generalized epilepsy. Additionally, no other psychiatric traits showed a causal relationship with epilepsy, including anxiety disorder, ASD, BIP, SCZ, and insomnia.

To our knowledge, various comorbidities are up to eight times more prevalent in people with epilepsy than in the general population (Thijs et al., 2019). Psychiatric illness is one of the most common comorbidities, with significant overrepresentation both in adults and in children of patients with epilepsy (Mula et al., 2021). Population‐based studies identified a 35% lifetime prevalence of psychiatric comorbidities before and after the diagnosis of epilepsy (Kanner, 2017). Nevertheless, these empirical statistical associations could not clearly clarify the essential relationship between psychiatric comorbidities and epilepsy. Understanding the causal effect has significant implications for early screening and treatment of corresponding psychiatric disorders, especially in patients with new‐onset epilepsy (Keezer et al., 2016). Besides, more undiscovered mechanisms are encouraged to be detected as new targets for effective and low‐risk drugs. Shuai et al. conducted an MR study to investigate modifiable risk factors for epilepsy, including depression. However, their study only enrolled one psychiatric trait, and the data on depression were limited by a small sample of 901 cases from the UK biobank (Yuan et al., 2021).

Consistent with previous observational, population‐based studies that showed an increased prevalence of depression in patients with epilepsy, our MR analysis determined a risk effect of MDD on epilepsy. The prevalence rate in patients with epilepsy was as high as 17%–22%, which was up to 55% in patients with drug‐resistant epilepsy (Tellez‐Zenteno et al., 2007). Moreover, the odds ratio for the risk of epilepsy in patients with MDD was 2.5 (Adelow et al., 2012). Another study based on the UK General Practice Research Database found that the incidence of depression is significantly higher during the three years preceding the development of epilepsy (Hesdorffer et al., 2012). All these results, to some extent, suggested that depression may increase the risk of the onset of epilepsy. In terms of seizure types, focal epilepsy was reported to be associated with a higher prevalence of depression than generalized epilepsy (Kim et al., 2018; Sanchez‐Gistau et al., 2012). Correspondingly, we found a causal effect of MDD only on focal epilepsy.

Several animal research studies found that many neurobiological pathogenic mechanisms of primary MDDs may potentially promote the development of seizures either spontaneously or with an insult to the central nervous system, such as endocrine abnormalities, structural and functional abnormalities of cortex, neurotransmitter abnormalities and immunological abnormalities (Singh & Goel, 2021; Kanner, 2011). First, patients with a primary MDD were found with high blood cortisol concentrations that may cause epileptogenesis. Second, patients with a primary MDD may have decreased cortical thickness, which play a part in worse seizure control. Third, abnormality of neurotransmitters, especially serotonin and norepinephrine, in patients with a primary MDD can increase the risk of epilepsy.

ADHD is common in children with epilepsy, with a prevalence from 12% to 39% in patients with newly diagnosed epilepsy and up to 70% in drug‐resistant epilepsy (Rheims & Auvin, 2021). Excluding children, ADHD symptoms also occur in 20−30% of adult patients with epilepsy (Ashjazadeh et al., 2019). The prevalence of ADHD in children with epilepsy is five to ten times higher than that in controls without epilepsy (Cohen et al., 2013; Wagner et al., 2021). A study of 91,605 children (<17 years of age) from the National Survey of Children's Health in America, including 977 children with epilepsy, found that the prevalence of ADHD was much higher than that in children without epilepsy (23% vs. 6%) (Adams & Claussen, 2022). Conversely, epilepsy occurs approximately 4 times more frequently in children with ADHD than in the general population. ADHD was composed of a clear predominance of the combined type (80%), which has been reported to be more common in patients with generalized epilepsy, consistent with the conclusion in our study (Rheims & Auvin, 2021). However, the mechanisms of the relationship between ADHD and epilepsy need to be illustrated in the future.

Anxiety disorder was the second most common comorbidity after depressive disorder in patients with epilepsy. In adult patients with epilepsy, prevalence estimates for anxiety disorder range from 11% to 50% (Hingray et al., 2021). However, our study showed no causal relation between anxiety disorders and epilepsy, the reason for which may be bias from insufficient associated IVs (only 4 SNPs with the threshold of 1 × 10^−5^) and the objective heterogeneity compared with depressive disorder. Although we found a protective effect of BIP on epilepsy, no statistically significant results were obtained in the confirmation test in FinnGen or the meta‐analysis, and we did not find any supportive reports in previous studies (Knott et al., 2015).

The strengths of the present study designed with MR analysis were as follows. First, we used genetic variants allocated randomly to identify the causal effect of exposure (psychiatric traits) on outcome (epilepsy), which could reduce conventional bias and avoid reverse causality because of these three basic assumptions (Davies & Holmes, 2018). Second, SNPs strongly associated with psychiatric traits and epilepsy were obtained from GWASs with large sample sizes, thereby increasing the reliability when interpreting the causal effect sizes of the results. Specially, the accurate selection of the valid SNPs is the foundation to meet the study assumptions and output a convincing conclusion. For example, SNPs with high linkage disequilibrium and SNPs associated with outcomes must be carefully excluded among the exposure‐related SNPs before subsequent MR calculation. Third, this study selected seven psychiatric traits involving mood disorders, anxiety disorders, behavior disorders and sleep disorders both in adults and children, aiming to completely illustrate the causal effect of psychiatric disorders. Fourth, the conclusion became more convincing by confirming our results with a different data set, meta‐analysis, several methods, and sensitivity tests. Additionally, we distinguished different types of epilepsy from the data set and assessed the causal effect on focal epilepsy and generalized epilepsy.

There were also some limitations in this study. First, we did not evaluate the resultant relation between psychiatric traits and epilepsy because no SNP strongly associated with epilepsy was accessible to produce a reliable conclusion. Future studies are necessary whenever valid SNPs associated with epilepsy are available from any newly published GWAS. Second, the causal effect of ADHD on epilepsy calculated from FinnGen data was not statistically significant, although the results of the meta‐analysis of ILAE and FinnGen were consistent with the ILAE consortium. Considering the bias of the unbalanced samples from FinnGen (*n*_case_ = 6260 and *n*_control_ = 176,107), we finally regarded ADHD as a risk factor for epilepsy according to the conclusion of the ILAE and meta‐analysis. Third, because the GWAS summary statistics of epilepsy used in this study were not stratified by onset age, we could not perform further MR analysis to evaluate the causal effect of psychiatric traits, especially ADHD, on epilepsy in different populations (adults or children when diagnosed). Finally, our MR analysis was derived from data of European participants, which may not represent the general population.

## 5 CONCLUSIONS

In summary, our study provides evidence on the risk effect of MDD and ADHD on epilepsy mainly in the European population. More studies need to be conducted to explore the mechanism and biological pathways from psychiatric traits to epilepsy.

## AUTHOR CONTRIBUTIONS

All authors contributed to the study conception and design. Data acquirement were performed by Gongfei Li, Meiqi Zheng, Xiao Liu, Jiechuan Ren, and Tingting Yu. Gongfei Li and Minghui Wang constructed the script based on R 4.1.3 and analyzed the data. The first draft of the manuscript was written by Gongfei Li and Qun Wang commented and revised the manuscript critically for important intellectual content. All authors read and approved the final manuscript.

## FUNDING

The study was financially supported by the National Key Technologies R&D Program of China (2022YFC2503800 to QW), and the Beijing Municipal Natural Science Foundation (Z200024 to YGW and QW).

## CONFLICT OF INTEREST STATEMENT

All authors declare that they have no competing interests.

### PEER REVIEW

The peer review history for this article is available at https://publons.com/publon/10.1002/brb3.2939.

## Supporting information

Figure S1. MR leave‐one‐out sensitivity analysis for MDD on epilepsy in ILAE.

Figure S2. MR leave‐one‐out sensitivity analysis for MDD on epilepsy in FinnGen.

Figure S3. MR leave‐one‐out sensitivity analysis for ADHD on epilepsy in ILAE.

Figure S4. MR leave‐one‐out sensitivity analysis for ADHD on epilepsy in FinnGen.

Figure S5. MR leave‐one‐out sensitivity analysis for BIP on epilepsy in ILAE.

Figure S6. MR leave‐one‐out sensitivity analysis for BIP on epilepsy in FinnGen.

Table S1. Description of GWAS phenotype for each trait.
Table S2. 7 Valid instrumental variables used for Mendelian randomization analysis of attention deficit hyperactivity disorder (Exposure) on epilepsy (Outcome) from ILAE data.
Table S3. 22 Valid instrumental variables used for Mendelian randomization analysis of autism spectrum disorder (Exposure) on epilepsy (Outcome) from ILAE data.
Table S4. 23 Valid instrumental variables used for Mendelian randomization analysis of major depressive disorder (Exposure) on epilepsy (Outcome) from ILAE data.
Table S5. 11 Valid instrumental variables used for Mendelian randomization analysis of bipolar disorder (Exposure) on epilepsy (Outcome) from ILAE data.
Table S6. 55 Valid instrumental variables used for Mendelian randomization analysis of schizophrenia (Exposure) on epilepsy (Outcome) from ILAE data.
Table S7. 29 Valid instrumental variables used for Mendelian randomization analysis of insomnia (Exposure) on epilepsy (Outcome) from ILAE data.
Table S8. 4 Valid instrumental variables used for Mendelian randomization analysis of anxiety disorder (Exposure) on epilepsy (Outcome) from ILAE data.
Table S9. 10 Valid instrumental variables used for Mendelian randomization analysis of attention deficit hyperactivity disorder (Exposure) on epilepsy (Outcome) from FinnGen data.
Table S10. 36 Valid instrumental variables used for Mendelian randomization analysis of MDD (Exposure) on epilepsy (Outcome) from FinnGen data.
Table S11. 14 Valid instrumental variables used for Mendelian randomization analysis of BIP (Exposure) on epilepsy (Outcome) from FinnGen data.
Table S12. Pleiotropy and heterogeneity test of the instrumental variables for psychiatric traits on epilepsy.

## Data Availability

The data that support the findings of this study are openly available at https://gwas.mrcieu.ac.uk.
