# Supplementary figures and images for: Causal effect of psychiatric disorders on epilepsy: A two‐sample Mendelian randomization study
Source: Brain Behav. 2023 Mar 1;13(4):e2939. doi: 10.1002/brb3.2939 (PMC10097067; doi:10.1002/brb3.2939)

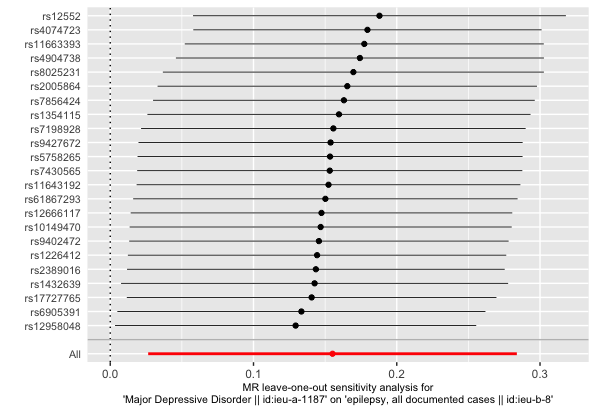

Supplement: Supplementary file 1 — Figure S1. MR leave‐one‐out sensitivity analysis for MDD on epilepsy in ILAE. [file BRB3-13-e2939-s003.tiff]

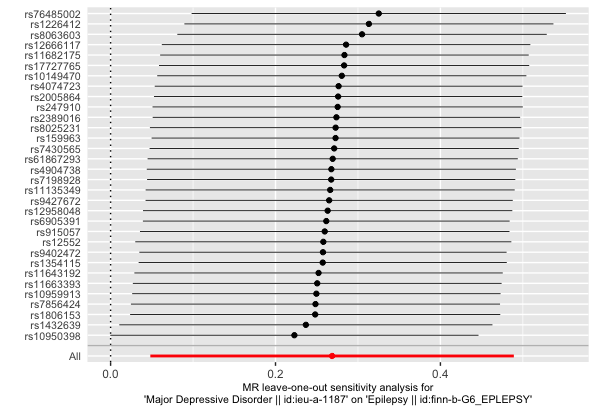

Supplement: Supplementary file 2 — Figure S2. MR leave‐one‐out sensitivity analysis for MDD on epilepsy in FinnGen. [file BRB3-13-e2939-s005.tiff]

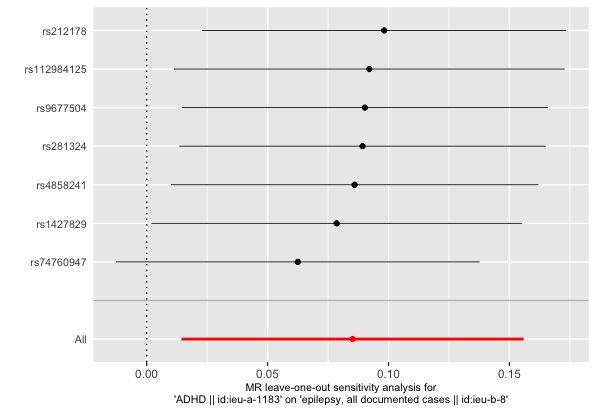

Supplement: Supplementary file 3 — Figure S3. MR leave‐one‐out sensitivity analysis for ADHD on epilepsy in ILAE. [file BRB3-13-e2939-s001.tiff]

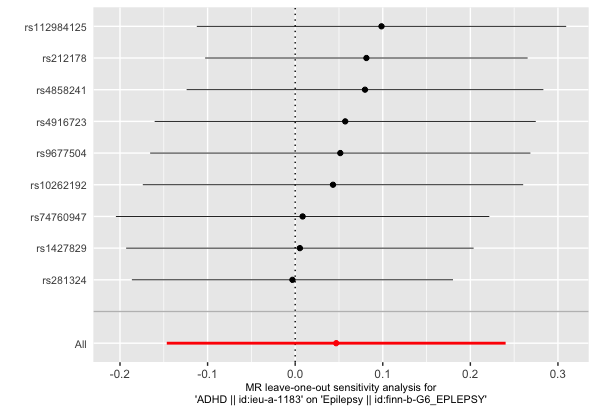

Supplement: Supplementary file 4 — Figure S4. MR leave‐one‐out sensitivity analysis for ADHD on epilepsy in FinnGen. [file BRB3-13-e2939-s006.tiff]

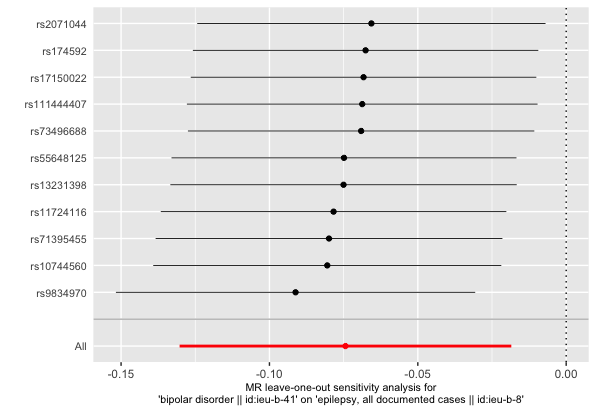

Supplement: Supplementary file 5 — Figure S5. MR leave‐one‐out sensitivity analysis for BIP on epilepsy in ILAE. [file BRB3-13-e2939-s007.tiff]

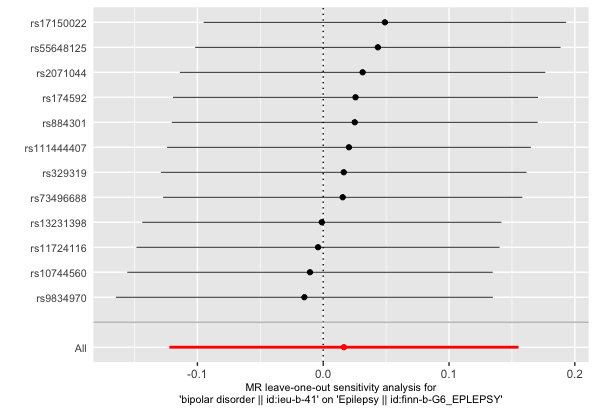

Supplement: Supplementary file 6 — Figure S6. MR leave‐one‐out sensitivity analysis for BIP on epilepsy in FinnGen. [file BRB3-13-e2939-s004.tiff]
