# Supplementary material for: Causal effect of psychiatric disorders on epilepsy: A two‐sample Mendelian randomization study
Source: Brain Behav. 2023 Mar 1;13(4):e2939. doi: 10.1002/brb3.2939 (PMC10097067; doi:10.1002/brb3.2939)
Supplement: Supplementary file 7 — Table S1. Description of GWAS phenotype for each trait. Table S2. 7 Valid instrumental variables used for Mendelian randomization analysis of attention deficit hyperactivity disorder (Exposure) on epilepsy (Outcome) from ILAE data. Table S3. 22 Valid instrumental variables used for Mendelian randomization analysis of autism spectrum disorder (Exposure) on epilepsy (Outcome) from ILAE data. Table S4. 23 Valid instrumental variables used for Mendelian randomization analysis of major depressive disorder (Exposure) on epilepsy (Outcome) from ILAE data. Table S5. 11 Valid instrumental variables used for Mendelian randomization analysis of bipolar disorder (Exposure) on epilepsy (Outcome) from ILAE data. Table S6. 55 Valid instrumental variables used for Mendelian randomization analysis of schizophrenia (Exposure) on epilepsy (Outcome) from ILAE data. Table S7. 29 Valid instrumental variables used for Mendelian randomization analysis of insomnia (Exposure) on epilepsy (Outcome) from ILAE data. Table S8. 4 Valid instrumental variables used for Mendelian randomization analysis of anxiety disorder (Exposure) on epilepsy (Outcome) from ILAE data. Table S9. 10 Valid instrumental variables used for Mendelian randomization analysis of attention deficit hyperactivity disorder (Exposure) on epilepsy (Outcome) from FinnGen data. Table S10. 36 Valid instrumental variables used for Mendelian randomization analysis of MDD (Exposure) on epilepsy (Outcome) from FinnGen data. Table S11. 14 Valid instrumental variables used for Mendelian randomization analysis of BIP (Exposure) on epilepsy (Outcome) from FinnGen data. Table S12. Pleiotropy and heterogeneity test of the instrumental variables for psychiatric traits on epilepsy. [file BRB3-13-e2939-s002.docx]

**Index of Supplementary Tables**

Table S1. Description of GWAS phenotype for each trait.

Table S2. 7 valid instrumental variables used for Mendelian randomization analysis of attention deficit hyperactivity disorder (Exposure) on epilepsy (Outcome) from ILAE data.

Table S3. 22 valid instrumental variables used for Mendelian randomization analysis of autism spectrum disorder (Exposure) on epilepsy (Outcome) from ILAE data.

Table S4. 23 valid instrumental variables used for Mendelian randomization analysis of major depressive disorder (Exposure) on epilepsy (Outcome) from ILAE data.

Table S5. 11 valid instrumental variables used for Mendelian randomization analysis of bipolar disorder (Exposure) on epilepsy (Outcome) from ILAE data.

Table S6. 55 valid instrumental variables used for Mendelian randomization analysis of schizophrenia (Exposure) on epilepsy (Outcome) from ILAE data.

Table S7. 29 valid instrumental variables used for Mendelian randomization analysis of insomnia (Exposure) on epilepsy (Outcome) from ILAE data.

Table S8. 4 valid instrumental variables used for Mendelian randomization analysis of anxiety disorder (Exposure) on epilepsy (Outcome) from ILAE data.

Table S9. 10 Valid instrumental variables used for Mendelian randomization analysis of attention deficit hyperactivity disorder (Exposure) on epilepsy (Outcome) from FinnGen data.

Table S10. 36 Valid instrumental variables used for Mendelian randomization analysis of MDD (Exposure) on epilepsy (Outcome) from FinnGen data.

Table S11. 14 Valid instrumental variables used for Mendelian randomization analysis of BIP (Exposure) on epilepsy (Outcome) from FinnGen data.

Table S12. Pleiotropy and heterogeneity test of the instrumental variables for psychiatric traits on epilepsy.

**Table S1. Definition of GWAS phenotype for each trait.**

| **Trait** | **GWAS Phenotype Definition** |
| --- | --- |
| ADHD | Cases were diagnosed by psychiatrists at in- or out-patient clinics, predominantly the latter according to ICD10 |
| ASD | Cases were those met from either the Autism Diagnostic Interview-Revised (ADI-R) or the Autism Diagnostic Observation Schedule (ADOS) domain scores. |
| MDD | Cases met international consensus criteria (DSM-V or ICD-10) for a lifetime diagnosis of MDD |
| BIP | Cases met international consensus criteria (DSM-V or ICD-10) for a lifetime diagnosis of BIP |
| Insomnia | Samples whose answer is “usually” to question “Do you have trouble falling asleep at night or do you wake up in the middle of the night?” will be included as a case. |
| SCZ | Cases validation acquired both clinical and consensus research diagnosis. |
| Anxiety disorder | Cases met one of two definitions. First was self-reporting a lifetime professional diagnosis of an anxiety disorder. Second was meeting criteria for a likely lifetime diagnosis of DSM-5 generalised anxiety disorder. |

ADHD, attention deficit hyperactivity disorder; ASD, autism spectrum disorder; MDD, major depressive disorder; BIP, bipolar disorder; SCZ, schizophrenia.

**Table S2. 7 valid instrumental variables used for Mendelian randomization analysis of attention deficit hyperactivity disorder (Exposure) on epilepsy (Outcome) from ILAE data.**

| **SNP** | **Effect allele** | **Non-effect allele** | **Beta** | **SE** | ***P*** |
| --- | --- | --- | --- | --- | --- |
| **rs112984125** | A | G | 0.106 | 0.015 | 3.58E-13 |
| **rs1427829** | G | A | 0.080 | 0.013 | 1.82E-09 |
| **rs212178** | A | G | 0.115 | 0.020 | 7.68E-09 |
| **rs281324** | C | T | 0.074 | 0.013 | 2.68E-08 |
| **rs4858241** | G | T | 0.079 | 0.014 | 1.74E-08 |
| **rs74760947** | G | A | 0.180 | 0.032 | 1.35E-08 |
| **rs9677504** | A | G | 0.117 | 0.021 | 1.39E-08 |

**Table S3. 22 valid instrumental variables used for Mendelian randomization analysis of autism spectrum disorder (Exposure) on epilepsy (Outcome) from ILAE data.**

| **SNP** | **Effect allele** | **Non-effect allele** | **Beta** | **SE** | ***P*** |
| --- | --- | --- | --- | --- | --- |
| **rs10099100** | C | G | 0.084 | 0.015 | 1.07E-08 |
| **rs10110094** | G | A | 0.091 | 0.019 | 2.05E-06 |
| **rs116346488** | A | G | 0.143 | 0.032 | 7.58E-06 |
| **rs1452075** | T | C | 0.081 | 0.016 | 2.07E-07 |
| **rs1522603** | C | T | 0.064 | 0.014 | 5.34E-06 |
| **rs1548635** | A | T | 0.062 | 0.014 | 8.45E-06 |
| **rs16879023** | A | G | 0.096 | 0.020 | 1.76E-06 |
| **rs2224274** | T | C | 0.071 | 0.014 | 2.86E-07 |
| **rs2391769** | G | A | 0.077 | 0.015 | 1.14E-07 |
| **rs28729902** | G | A | 0.084 | 0.018 | 2.34E-06 |
| **rs325485** | G | A | 0.073 | 0.014 | 3.25E-07 |
| **rs34509057** | A | G | 0.073 | 0.017 | 9.73E-06 |
| **rs35404050** | T | C | 0.084 | 0.018 | 1.61E-06 |
| **rs41363353** | G | C | 0.084 | 0.019 | 8.61E-06 |
| **rs4609618** | C | A | 0.064 | 0.014 | 9.34E-06 |
| **rs6430841** | G | A | 0.080 | 0.018 | 6.40E-06 |
| **rs7578456** | G | A | 0.064 | 0.014 | 6.53E-06 |
| **rs7783557** | C | T | 0.067 | 0.015 | 4.36E-06 |
| **rs78058104** | A | G | 0.188 | 0.040 | 2.22E-06 |
| **rs910805** | A | G | 0.096 | 0.016 | 2.04E-09 |
| **rs9366877** | G | A | 0.068 | 0.014 | 9.05E-07 |
| **rs9389208** | T | C | 0.067 | 0.014 | 3.12E-06 |

**Table S4. 24 valid instrumental variables used for Mendelian randomization analysis of major depressive disorder (Exposure) on epilepsy (Outcome) from ILAE data.**

| **SNP** | **Effect allele** | **Non-effect allele** | **Beta** | **SE** | ***P*** |
| --- | --- | --- | --- | --- | --- |
| **rs10149470** | G | A | 0.029 | 0.005 | 3.05E-09 |
| **rs11643192** | A | C | 0.027 | 0.005 | 3.36E-08 |
| **rs11663393** | A | G | 0.028 | 0.005 | 1.65E-08 |
| **rs1226412** | T | C | 0.033 | 0.006 | 2.38E-08 |
| **rs12552** | G | A | 0.043 | 0.005 | 6.07E-19 |
| **rs12666117** | A | G | 0.027 | 0.005 | 1.35E-08 |
| **rs12958048** | G | A | 0.034 | 0.005 | 3.61E-11 |
| **rs1354115** | A | C | 0.028 | 0.005 | 2.37E-08 |
| **rs1432639** | A | C | 0.039 | 0.005 | 4.55E-15 |
| **rs17727765** | C | T | 0.051 | 0.009 | 8.51E-09 |
| **rs2005864** | T | C | 0.028 | 0.005 | 6.73E-09 |
| **rs2389016** | T | C | 0.031 | 0.005 | 1.02E-08 |
| **rs4074723** | C | A | 0.027 | 0.005 | 3.12E-08 |
| **rs4904738** | C | T | 0.029 | 0.005 | 2.57E-09 |
| **rs5758265** | A | G | 0.031 | 0.005 | 7.55E-09 |
| **rs61867293** | T | C | 0.037 | 0.006 | 6.97E-10 |
| **rs6905391** | A | G | 0.044 | 0.007 | 1.35E-10 |
| **rs7198928** | C | T | 0.028 | 0.005 | 1.00E-08 |
| **rs7430565** | A | G | 0.029 | 0.005 | 2.87E-09 |
| **rs7856424** | T | C | 0.031 | 0.005 | 8.48E-09 |
| **rs8025231** | C | A | 0.034 | 0.005 | 2.36E-12 |
| **rs9402472** | A | G | 0.033 | 0.006 | 2.78E-08 |
| **rs9427672** | G | A | 0.032 | 0.006 | 3.12E-08 |

**Table S5. 13 valid instrumental variables used for Mendelian randomization analysis of bipolar disorder (Exposure) on epilepsy (Outcome) from ILAE data.**

| **SNP** | **Effect allele** | **Non-effect allele** | **Beta** | **SE** | ***P*** |
| --- | --- | --- | --- | --- | --- |
| **rs10744560** | T | C | 0.083 | 0.014 | 2.92E-09 |
| **rs111444407** | T | C | 0.117 | 0.018 | 2.40E-10 |
| **rs11724116** | T | C | 0.104 | 0.019 | 3.27E-08 |
| **rs13231398** | C | G | 0.121 | 0.022 | 3.36E-08 |
| **rs17150022** | C | T | 0.113 | 0.020 | 2.70E-08 |
| **rs174592** | G | A | 0.077 | 0.014 | 3.66E-08 |
| **rs2071044** | T | C | 0.078 | 0.014 | 9.09E-09 |
| **rs55648125** | G | A | 0.117 | 0.022 | 4.92E-08 |
| **rs71395455** | G | A | 0.082 | 0.015 | 1.93E-08 |
| **rs73496688** | A | T | 0.109 | 0.019 | 1.05E-08 |
| **rs9834970** | C | T | 0.101 | 0.013 | 5.53E-14 |

**Table S6. 55 valid instrumental variables used for Mendelian randomization analysis of schizophrenia (Exposure) on epilepsy (Outcome) from ILAE data.**

| **SNP** | **Effect allele** | **Non-effect allele** | **Beta** | **SE** | ***P*** |
| --- | --- | --- | --- | --- | --- |
| **rs10108725** | T | C | 0.073 | 0.013 | 3.32E-08 |
| **rs10791097** | G | T | 0.077 | 0.011 | 2.05E-12 |
| **rs11027857** | A | G | 0.064 | 0.011 | 3.67E-09 |
| **rs1106568** | A | G | 0.069 | 0.013 | 2.85E-08 |
| **rs111294930** | G | A | 0.088 | 0.014 | 9.29E-10 |
| **rs11139497** | A | T | 0.066 | 0.012 | 2.65E-08 |
| **rs11191419** | A | T | 0.102 | 0.012 | 6.69E-18 |
| **rs11210892** | A | G | 0.068 | 0.012 | 3.42E-09 |
| **rs11682175** | C | T | 0.073 | 0.011 | 1.58E-11 |
| **rs11693094** | T | C | 0.074 | 0.011 | 2.17E-11 |
| **rs11693528** | G | C | 0.103 | 0.014 | 4.73E-14 |
| **rs117074560** | T | C | 0.157 | 0.028 | 1.66E-08 |
| **rs12691307** | G | A | 0.072 | 0.011 | 2.03E-10 |
| **rs12704290** | A | G | 0.106 | 0.017 | 2.59E-10 |
| **rs12887734** | T | G | 0.088 | 0.012 | 3.72E-13 |
| **rs12932476** | G | C | 0.060 | 0.011 | 4.62E-08 |
| **rs13240464** | C | T | 0.081 | 0.012 | 3.12E-12 |
| **rs1498232** | C | T | 0.072 | 0.012 | 1.21E-09 |
| **rs1615350** | T | C | 0.085 | 0.012 | 4.26E-12 |
| **rs16867576** | G | A | 0.096 | 0.017 | 1.60E-08 |
| **rs1702294** | C | T | 0.118 | 0.014 | 1.03E-17 |
| **rs17108967** | C | T | 0.065 | 0.012 | 1.21E-08 |
| **rs17194490** | T | G | 0.097 | 0.015 | 6.38E-11 |
| **rs2053079** | G | A | 0.072 | 0.013 | 1.74E-08 |
| **rs2103655** | A | G | 0.077 | 0.012 | 1.24E-10 |
| **rs215411** | A | T | 0.069 | 0.012 | 1.68E-09 |
| **rs2332700** | G | C | 0.077 | 0.013 | 7.38E-10 |
| **rs2414718** | A | G | 0.070 | 0.011 | 1.98E-10 |
| **rs2535627** | C | T | 0.070 | 0.011 | 1.17E-10 |
| **rs2693698** | G | A | 0.062 | 0.011 | 2.99E-08 |
| **rs28681284** | T | C | 0.102 | 0.014 | 6.35E-13 |
| **rs2905432** | A | G | 0.066 | 0.011 | 7.51E-09 |
| **rs2909457** | A | G | 0.060 | 0.011 | 4.25E-08 |
| **rs301797** | A | C | 0.066 | 0.012 | 1.20E-08 |
| **rs34796896** | A | G | 0.082 | 0.014 | 1.23E-09 |
| **rs35324223** | G | A | 0.092 | 0.015 | 2.04E-10 |
| **rs35998080** | T | G | 0.069 | 0.011 | 6.95E-10 |
| **rs36068923** | G | A | 0.084 | 0.013 | 4.14E-10 |
| **rs3849046** | T | C | 0.062 | 0.011 | 1.04E-08 |
| **rs4129585** | C | A | 0.079 | 0.011 | 3.61E-13 |
| **rs4391122** | G | A | 0.078 | 0.011 | 8.90E-13 |
| **rs4523957** | T | G | 0.070 | 0.012 | 1.40E-09 |
| **rs58120505** | C | T | 0.082 | 0.011 | 1.26E-13 |
| **rs5995756** | C | T | 0.072 | 0.011 | 2.91E-11 |
| **rs6439649** | T | G | 0.071 | 0.011 | 1.37E-10 |
| **rs6704768** | A | G | 0.077 | 0.011 | 2.06E-12 |
| **rs73229090** | A | C | 0.100 | 0.018 | 1.95E-08 |
| **rs75968099** | T | C | 0.080 | 0.011 | 2.31E-12 |
| **rs7601312** | G | A | 0.059 | 0.011 | 4.67E-08 |
| **rs7801375** | G | A | 0.083 | 0.015 | 2.88E-08 |
| **rs783540** | G | A | 0.060 | 0.011 | 4.77E-08 |
| **rs7893279** | G | T | 0.112 | 0.018 | 1.24E-10 |
| **rs8055219** | A | G | 0.077 | 0.013 | 1.45E-09 |
| **rs832190** | T | C | 0.070 | 0.011 | 5.73E-10 |
| **rs9636107** | G | A | 0.080 | 0.011 | 2.17E-13 |

**Table S7. 29 valid instrumental variables used for Mendelian randomization analysis of insomnia (Exposure) on epilepsy (Outcome) from ILAE data.**

| **SNP** | **Effect allele** | **Non-effect allele** | **Beta** | **SE** | ***P*** |
| --- | --- | --- | --- | --- | --- |
| **rs10838708** | A | G | 0.009 | 0.002 | 2.90E-10 |
| **rs11097861** | G | A | 0.010 | 0.002 | 1.10E-09 |
| **rs11152363** | A | G | 0.016 | 0.002 | 4.50E-16 |
| **rs11635495** | C | T | 0.009 | 0.001 | 2.80E-10 |
| **rs11790060** | C | T | 0.010 | 0.002 | 5.80E-11 |
| **rs12049261** | C | G | 0.011 | 0.002 | 6.80E-12 |
| **rs12470989** | G | A | 0.010 | 0.002 | 2.80E-08 |
| **rs1430205** | T | C | 0.009 | 0.001 | 2.10E-10 |
| **rs1592757** | C | G | 0.010 | 0.002 | 4.30E-11 |
| **rs17151854** | T | G | 0.013 | 0.002 | 3.80E-10 |
| **rs17709610** | G | A | 0.010 | 0.002 | 9.50E-10 |
| **rs1988337** | G | A | 0.008 | 0.001 | 2.10E-08 |
| **rs2014830** | T | C | 0.012 | 0.002 | 8.90E-13 |
| **rs224032** | A | G | 0.008 | 0.001 | 1.80E-08 |
| **rs2644128** | G | C | 0.011 | 0.001 | 1.00E-12 |
| **rs314280** | G | A | 0.010 | 0.001 | 7.30E-11 |
| **rs56093896** | A | C | 0.012 | 0.002 | 7.70E-12 |
| **rs56330606** | G | A | 0.009 | 0.002 | 1.20E-09 |
| **rs56365214** | A | C | 0.015 | 0.002 | 5.60E-13 |
| **rs6561715** | A | T | 0.012 | 0.002 | 4.80E-14 |
| **rs6690017** | G | T | 0.010 | 0.002 | 1.10E-11 |
| **rs68094047** | T | C | 0.010 | 0.002 | 1.70E-09 |
| **rs6975972** | G | A | 0.009 | 0.002 | 2.00E-09 |
| **rs705219** | A | T | 0.013 | 0.002 | 1.20E-08 |
| **rs7711696** | T | G | 0.011 | 0.002 | 4.10E-12 |
| **rs8180817** | C | G | 0.010 | 0.002 | 2.70E-11 |
| **rs9570080** | C | T | 0.011 | 0.002 | 1.60E-11 |
| **rs9845387** | A | C | 0.022 | 0.004 | 7.10E-09 |
| **rs9894577** | A | G | 0.013 | 0.002 | 1.30E-16 |

**Table S8. 4 valid instrumental variables used for Mendelian randomization analysis of anxiety disorder (Exposure) on epilepsy (Outcome) from ILAE data.**

| **SNP** | **Effect allele** | **Non-effect allele** | **Beta** | **SE** | ***P*** |
| --- | --- | --- | --- | --- | --- |
| **rs2294619** | G | A | 0.001 | 0.0003 | 6.50E-06 |
| **rs242077** | C | T | 0.001 | 0.0002 | 3.20E-06 |
| **rs71409272** | A | G | 0.002 | 0.0005 | 6.90E-07 |
| **rs7637829** | A | T | 0.001 | 0.0003 | 6.90E-06 |

**Table S9. 10 Valid instrumental variables used for Mendelian randomization analysis of attention deficit hyperactivity disorder (Exposure) on epilepsy (Outcome) from FinnGen data.**

| **SNP** | **Effect allele** | **Non-effect allele** | **Beta** | **SE** | ***P*** |
| --- | --- | --- | --- | --- | --- |
| **rs10262192** | A | G | **0.073** | **0.013** | **2.89E-08** |
| **rs112984125** | A | G | **0.106** | **0.015** | **3.58E-13** |
| **rs11591402** | A | T | **0.093** | **0.016** | **1.34E-08** |
| **rs1427829** | G | A | **0.080** | **0.013** | **1.82E-09** |
| **rs212178** | A | G | **0.115** | **0.020** | **7.68E-09** |
| **rs281324** | C | T | **0.074** | **0.013** | **2.68E-08** |
| **rs4858241** | G | T | **0.079** | **0.014** | **1.74E-08** |
| **rs4916723** | C | A | **0.077** | **0.014** | **1.58E-08** |
| **rs74760947** | G | A | **0.180** | **0.032** | **1.35E-08** |
| **rs9677504** | A | G | **0.117** | **0.021** | **1.39E-08** |

**Table S10. 36 Valid instrumental variables used for Mendelian randomization analysis of MDD (Exposure) on epilepsy (Outcome) from FinnGen data.**

| **SNP** | **Effect allele** | **Non-effect allele** | **Beta** | **SE** | ***P*** |
| --- | --- | --- | --- | --- | --- |
| **rs10149470** | G | A | 0.029 | 0.005 | 3.05E-09 |
| **rs10950398** | A | G | 0.027 | 0.005 | 2.55E-08 |
| **rs10959913** | G | T | 0.033 | 0.006 | 5.06E-09 |
| **rs11135349** | C | A | 0.029 | 0.005 | 1.09E-09 |
| **rs11643192** | A | C | 0.027 | 0.005 | 3.36E-08 |
| **rs11663393** | A | G | 0.028 | 0.005 | 1.65E-08 |
| **rs11682175** | C | T | 0.028 | 0.005 | 4.68E-09 |
| **rs1226412** | T | C | 0.033 | 0.006 | 2.38E-08 |
| **rs12552** | G | A | 0.043 | 0.005 | 6.07E-19 |
| **rs12666117** | A | G | 0.027 | 0.005 | 1.35E-08 |
| **rs12958048** | G | A | 0.034 | 0.005 | 3.61E-11 |
| **rs1354115** | A | C | 0.028 | 0.005 | 2.37E-08 |
| **rs1363104** | G | C | 0.031 | 0.005 | 7.38E-11 |
| **rs1432639** | A | C | 0.039 | 0.005 | 4.55E-15 |
| **rs159963** | A | C | 0.027 | 0.005 | 3.19E-08 |
| **rs17727765** | C | T | 0.051 | 0.009 | 8.51E-09 |
| **rs1806153** | T | G | 0.036 | 0.006 | 1.18E-09 |
| **rs2005864** | T | C | 0.028 | 0.005 | 6.73E-09 |
| **rs2389016** | T | C | 0.031 | 0.005 | 1.02E-08 |
| **rs247910** | G | A | 0.032 | 0.005 | 1.07E-10 |
| **rs34215985** | G | C | 0.037 | 0.006 | 3.13E-09 |
| **rs4074723** | C | A | 0.027 | 0.005 | 3.12E-08 |
| **rs4904738** | C | T | 0.029 | 0.005 | 2.57E-09 |
| **rs61867293** | T | C | 0.037 | 0.006 | 6.97E-10 |
| **rs62099069** | T | A | 0.028 | 0.005 | 1.31E-08 |
| **rs6905391** | A | G | 0.044 | 0.007 | 1.35E-10 |
| **rs7198928** | C | T | 0.028 | 0.005 | 1.00E-08 |
| **rs7430565** | A | G | 0.029 | 0.005 | 2.87E-09 |
| **rs76485002** | G | A | 0.109 | 0.018 | 1.60E-09 |
| **rs7856424** | T | C | 0.031 | 0.005 | 8.48E-09 |
| **rs8025231** | C | A | 0.034 | 0.005 | 2.36E-12 |
| **rs8063603** | A | G | 0.031 | 0.005 | 6.86E-09 |
| **rs915057** | G | A | 0.030 | 0.005 | 7.61E-10 |
| **rs9402472** | A | G | 0.033 | 0.006 | 2.78E-08 |
| **rs9402472** | A | G | 0.033 | 0.006 | 2.78E-08 |
| **rs9427672** | G | A | 0.032 | 0.006 | 3.12E-08 |

**Table S11. 14 Valid instrumental variables used for Mendelian randomization analysis of BIP (Exposure) on epilepsy (Outcome) from FinnGen data.**

| **SNP** | **Effect allele** | **Non-effect allele** | **Beta** | **SE** | ***P*** |
| --- | --- | --- | --- | --- | --- |
| **rs10744560** | T | C | 0.083 | 0.014 | 2.92E-09 |
| **rs111444407** | T | C | 0.117 | 0.018 | 2.40E-10 |
| **rs11724116** | T | C | 0.104 | 0.019 | 3.27E-08 |
| **rs13231398** | C | G | 0.121 | 0.022 | 3.36E-08 |
| **rs17150022** | C | T | 0.113 | 0.020 | 2.70E-08 |
| **rs174592** | G | A | 0.077 | 0.014 | 3.66E-08 |
| **rs2071044** | T | C | 0.078 | 0.014 | 9.09E-09 |
| **rs2314398** | G | C | 0.084 | 0.014 | 5.92E-09 |
| **rs329319** | G | A | 0.079 | 0.014 | 1.54E-08 |
| **rs55648125** | G | A | 0.117 | 0.022 | 4.92E-08 |
| **rs5758065** | G | C | 0.074 | 0.014 | 3.23E-08 |
| **rs73496688** | A | T | 0.109 | 0.019 | 1.05E-08 |
| **rs884301** | T | C | 0.080 | 0.014 | 5.80E-09 |
| **rs9834970** | C | T | 0.101 | 0.013 | 5.53E-14 |

**Table S12. Pleiotropy and heterogeneity test of the instrumental variables for psychiatric traits on epilepsy.**

| **Exposure** | **Outcome** | **MR-PRESSO global Test** | ***P*** | **Cochran’s *Q*** | **df** | ***P*** |
| --- | --- | --- | --- | --- | --- | --- |
| ADHD | Epilepsy | 5.17 | 0.707 | 3.48 | 5 | 0.627 |
| ASD |  | 47.62 | 0.006 | 35.46 | 17 | 0.005 |
| MDD |  | 41.46 | 0.025 | 32.26 | 21 | 0.055 |
| BIP |  | 8.25 | 0.864 | 5.29 | 9 | 0.808 |
| SCZ |  | 118.18 | <0.001 | 111.95 | 52 | 2.80×10^-6^ |
| insomnia |  | 34.98 | 0.245 | 31.90 | 25 | 0.161 |
| anxiety disorder |  | 7.37 | 0.294 | 1.39 | 2 | 0.499 |

ADHD, attention deficit/hyperactivity disorder; ASD, autism spectrum disorder; BIP, bipolar disorder; MDD, major depressive disorder; SCZ, schizophrenia; MR-PRESSO, mendelian randomization pleiotropy residual sum and outlier.
